# The effect of gonadotrophin-releasing hormone agonist versus human chorionic gonadotrophin trigger on pregnancy and neonatal outcomes in Letrozole-HMG IUI cycles

**DOI:** 10.1186/s12884-023-05835-8

**Published:** 2023-07-13

**Authors:** Li Chen, Qianwen Xi, Shutian Jiang, Yining Gao, Hui Long, Yao Wang, Yanping Kuang

**Affiliations:** Department of Assisted Reproduction, Shanghai Ninth People’s Hospital, Shanghai Jiao Tong University School of Medicine, 639 Zhizaoju Road, Shanghai, 200011 China

**Keywords:** Intrauterine insemination, Letrozole, Ovulation trigger, Gonadotrophin-releasing hormone agonist, Human chorionic gonadotrophin, Clinical pregnancy rate, Infertility

## Abstract

**Background:**

GnRHa and hCG are both used for oocyte maturation and ovulation triggering. However, GnRHa have a shorter half-life than hCG, which leads to luteal phase deficiency. Letrozole (LE) has been found to improve the luteal function. Thus, the choice of triggering strategy can be different in intrauterine insemination (IUI) cycles using LE and human menopausal gonadotropin (HMG). The aim of this study was to compare the pregnancy and neonatal outcomes of patients triggered with GnRHa versus hCG versus dual trigger in LE-IUI cycles.

**Methods:**

This retrospective cohort study included 6,075 LE-HMG IUI cycles between January 2010 and May 2021 at a tertiary-care academic medical center in China. All cycles were divided into three groups according to different trigger strategies as hCG trigger group, GnRHa trigger group and dual trigger group. The primary outcome was clinical pregnancy rate. Logistic regression analysis was performed to explore other risk factors for clinical pregnancy rate.

**Results:**

No significant difference was observed in clinical pregnancy rate between hCG, GnRHa and dual trigger cycles in LE-HMG IUI cycles (*P* = 0.964). The miscarriage rate was significantly lower in the GnRHa trigger group, and higher in the dual trigger group, compared with the hCG group (*P* = 0.045). Logistic analysis confirmed that triggering strategy was associated with miscarriage (aOR:0.427, 95%CI: 0.183–0.996, *P* = 0.049; aOR:0.298, 95%CI: 0.128–0.693, *P* = 0.005). No significant differences were observed regarding neonatal outcomes between the three groups.

**Conclusions:**

Our findings suggested that both GnRHa and dual trigger can be used to trigger ovulation in LE-HMG IUI cycles, but dual trigger must be used with caution.

## Background

Intrauterine insemination (IUI) with ovulation triggering has been widely used for infertility treatment. The reported clinical pregnancy rate varies, ranging from 5 to 20% [1–3]. The outcome of IUI depends on many factors. Among them, the correct timing of insemination is one of the most important ones [4]. Triggering ovulation is commonly used for the timing of IUI.

In IUI cycles, human chorionic gonadotrophin (hCG) and gonadotrophin-releasing hormone agonist (GnRHa) are frequently used for oocytes maturation and ovulation triggering. Another choice is to use dual trigger, a combination of both GnRHa and hCG [5]. hCG is used as a surrogate for the natural LH surge, while a single bolus injection of GnRHa can induce both endogenous LH and FSH surge due to the initial flare effect [6]. The LH surge is known to stimulate arrested oocytes to resume meiosis [7]. The FSH peak can also promote the resumption of meiosis in addition to cumulus expansion, and oocyte nuclear maturation [8]. Inducing both the LH and FSH surge at the same time makes GnRHa a more physiological approach for inducing oocytes maturation. However, compared with hCG, GnRHa has a much shorter half-life, which reduces its stimulation of the corpora luteum [9] and leads to luteal phase deficiency [10, 11]. Luteal phase support has been proven effective for rescuing corpus luteum function and improving pregnancy rates in GnRHa-triggered cycles [12–14], indicating the situation of compromised luteal functions in cycles triggered with GnRHa. Dual trigger can also solve these problems, but it is necessary to consider whether the advantages outweigh the inconvenience and cost of using both drugs.

Several studies have been done to compare GnRHa with hCG trigger in IUI cycles, but the findings are inconsistent [15–17]. In a study by Romeu et al., significant difference was founded in pregnancy rates between GnRHa and hCG group (27.25% vs 17.31%, *P* = 0.0007) [15]. Another study conducted by Soliman et al. also reported higher clinical pregnancy rate in the GnRHa-trigger group than that in the hCG group (17.61% vs. 13.06%), but the result was not significant [16]. Le et al. observed no significant difference either, but they found higher pregnancy rate in the hCG-trigger group instead of the GnRHa-triggrt group (23.2% vs. 13.3%) [17]. Of note, almost all of them used either clomiphene citrate (CC) or human menopausal gonadotropin (HMG) for ovulation stimulation [16–18].

In the last two decades, letrozole (LE) was increasingly being used as an ovulation stimulation drug [19]. It is a highly potent, reversible aromatase inhibitor, which shows its superiority of no adverse effect on cervical mucus and endometrial morphology, due to its lack of antiestrogenic effect [19]. In addition, a study by Liu et al. [20] showed that higher pregnancy and live birth rates were achieved when patients were given LE combined with HMG in comparison with natural cycles, CC, LE, HMG, or CC combined with HMG. More importantly, LE has been found to improve the luteal function through better stimulation of the corpus luteum and progesterone secretion [21], which can potentially be used to rescue the corpus luteum function and improve pregnancy outcomes in GnRHa-triggered cycles. CC can be used as a therapy for luteal phase defect during the early luteal phase. However, many studies have shown that the incidence of luteal phase defect after induction of ovulation with CC is significant, ranging from 19 to 50% [22–24]. Thus, the choice of triggering strategy can be different in LE-HMG IUI cycles compared with CC cycles. Our study is among the first to investigate the effect of triggering strategy in LE-HMG IUI cycles with a large sample size.

The purpose of the present study is to compare the pregnancy and neonatal outcomes of patients triggered with GnRHa versus hCG versus dual trigger in LE-HMG IUI cycles.

## Methods

This retrospective cohort study was conducted at the Department of Assisted Reproduction of the Ninth People's Hospital of Shanghai Jiao Tong University School of Medicine between January 2010 to May 2021. It was approved by the Ethics Committee (Institutional Review Board) of Shanghai Ninth People's Hospital. Written informed consent was waived due to the retrospective nature by the Ethics Committee (Institutional Review Board) of Shanghai Ninth People's Hospital, and the patient's data was used anonymously.

Basic characteristics of the patients, including menstrual cycle (MC) day3 follicle-stimulating hormone (FSH), luteinizing hormone (LH), estradiol (E2), progesterone (P), prolactin, anti-Müllerian hormone (AMH), transvaginal ultrasound (TVS) of the pelvis, and hysteroscope for the female partner, were investigated. Semen analysis parameters of their male partners were also collected. Couples who meet the following criteria are included: [1] age no older than 40, [2] patent fallopian tubes and normal uterine cavity according to hysteroscope, [3] sperm concentration of male partners > 10 million sperm/ml. In addition, patients also need to meet one of the following infertility causes: ovulatory dysfunction, unexplained factor, male factor and sexual dysfunction.

Only the first three cycles were recruited if one went through more than three cycles in the period. Cycles with incomplete medical records or stimulating protocols without LE and HMG were excluded.

Patients were given 2.5 mg/5 mg oral LE (Jiangsu Hengrui Medicine Co., China) once a day for 3–5 days since day 3 of menstruation, after ultrasound screen and blood test confirmed the presence of a baseline hormone profile. The LE dosage and duration were determined according to the patient’s length of menstrual cycle (MC) and BMI. The longer the menstrual cycle and the greater the BMI value, the longer duration and the larger dosage of LE were prescribed. Follicular monitoring started on MC10 and was carried out every 2 days by transvaginal ultrasound examination to record the development of follicles. On the same days, serum FSH, LH, E2, and P values were measured using blood tests. If the leading follicle size was not greater than 14 mm on MC10, 75 IU HMG (Anhui Fengyuan Pharmaceutical Co., China) was added. The duration of HMG varied according to the follicle response. Once a lead follicle reached a mean diameter of 18 mm or a slightly elevated LH was observed (10mIU/ml < LH ≤ 15 mIU/ml) when the lead follicle reached a mean diameter of 16 mm, hCG (5,000 IU) (Lizhu Pharmaceutical Trading Co.), triptorelin (0.1 mg; Decapeptyl, Ferring Pharmaceuticals) or a combination of triptorelin (0.1 mg) and hCG (5,000 IU)was administered to induce ovulation. The choice of the specific trigger strategy was based on the doctor’s preference. This day was called "trigger day". If a markedly elevated LH (LH > 15 mIU/ml) occurred, IUI will be performed within 24 h. If there were more than 3 dominant follicles on trigger day, the cycle would be canceled.

After 2–3 days of abstinence, semen samples were obtained through masturbation, and then liquefied for 15–20 min. After that, the sample was washed with 3-layer density gradient centrifugation using Isolate (Irvine Scientific, USA). Insemination was performed 38–41 h after the trigger with a soft catheter (Cook Group, USA). Only one insemination was performed in one cycle.

The primary outcome of the study was clinical pregnancy rate (CPR). Secondary outcomes included biochemical pregnancy rate, multiple pregnancy rate, ongoing pregnancy rate, live birth rate, miscarriage rate, ectopic pregnancy rate, and neonatal indicators (birth weight, birth defect, etc.). All outcomes were defined according to WHO.

According to different triggering strategies, all cycles were divided into three groups: hCG trigger group, GnRHa trigger group, and dual trigger group.

Statistical analysis was performed with the Statistical Package for the Social Sciences (version 26.0; SPSS Inc., USA). Continuous variables were presented as mean ± SD. Normality of continuous variables was determined using Shapiro–Wilk test and illustration of Q-Q plots. The variables were compared by one-way analysis of variance (ANOVA) with post-hoc analysis or Welch's t-test as required. Categorical variables were expressed as number of cases (n) with rate (%). The variables were compared between groups by Chi-square test or Fisher's exact test as required. Binary logistic regression analysis was used to evaluate other factors' impact on CPR. *P* < 0.05 was considered statistically significant.

## Results

A total of 4,910 patients undergoing 6,075 IUI cycles from January 2010 to May 2021 were included in this study. Among them, 1,568 (25.8%) cycles were sorted into the hCG trigger group; 4,034 (66.4%) cycles were sorted into the GnRHa trigger group; the remaining cycles were classified as the dual trigger group.

The baseline characteristics and hormonal profiles of the patients are presented in Table 1. No significant differences were observed regarding the female age, male age, female BMI, male BMI, duration of infertility, or infertility type between the three groups. Meanwhile, the antral follicle count (AFC) was significantly higher in the dual trigger group. For the basal hormone profiles, the group triggered by GnRHa revealed significantly higher E2 (*P* = 0.012) and P (*P* = 0.002) than the hCG trigger group. The basal FSH, LH, and AMH were all comparable. Differences could also be observed in the distribution of infertility causes and rank of IUI attempts among the three groups.

**Table 1:** The demographic and basic characteristics of patients

	Group A: 5000 IU hCG (*n* = 1264)	Group B: 0.1 mg GnRHa (*n* = 3265)	Group C: 5000 IU hCG + 0.1 mg GnRHa (*n* = 381)	*P* value
Female age (years)	31.06 ± 3.68	31.06 ± 3.59	31.28 ± 3.63	0.542
Male age (years)	32.79 ± 4.57	32.89 ± 4.40	32.92 ± 4.73	0.800
Female BMI (kg/m^2^)	22.41 ± 3.47	22.09 ± 5.24	22.28 ± 3.43	0.109
Male BMI (kg/m^2^)	24.67 ± 3.54	24.50 ± 3.41	24.62 ± 3.47	0.351
Duration of infertility (years)	3.12 ± 2.12	3.13 ± 2.05	3.37 ± 2.40	0.161
Infertility type, n (%)				0.068
Primary	893 (70.6)	2191 (67.1)	256 (67.2)	
Secondary	371 (29.4)	1074 (32.9)	125 (32.8)	
Infertility causes, n(%)				< 0.001
Ovulatory dysfunction	255 (20.2)	622 (19.1)	83 (21.8)	
Sexual dysfunction	93 (7.4)	127 (3.9)	31 (8.1)	
Male factor	175 (13.8)	484 (14.8)	62 (16.3)	
Unexplained factor	741 (58.6)	2032 (62.2)	205 (53.8)	
Antral follicles	13.77 ± 6.75	13.55 ± 6.47	16.05 ± 6.55	< 0.001^a^
Basal hormonal level				
FSH (mIU/mL)	5.64 ± 1.28	5.62 ± 1.32	5.63 ± 1.32	0.874
LH (mIU/mL)	4.00 ± 2.21	4.00 ± 2.32	4.05 ± 1.91	0.903
E_2_ (pg/mL)	33.21 ± 14.72	34.79 ± 15.33	34.36 ± 14.38	0.012^b^
P (ng/mL)	0.27 ± 0.13	0.28 ± 0.14	0.25 ± 0.13	0.002^c^
AMH (ng/mL)	4.66 ± 4.15	4.60 ± 3.65	5.85 ± 5.27	0.542
Rank of IUI attempts, n (%)				< 0.001
1^st^ cycle	619 (49.0)	1717 (52.6)	165 (43.3)	
2^nd^ cycle	494 (39.1)	1259 (38.6)	187 (49.1)	
3^rd^ cycle	151 (11.9)	289 (8.9)	29 (7.6)	

*Abbreviations*: *BMI* body mass index, *FSH* follicle-stimulating hormone, *LH* luteinizing hormone, *E*_*2*_ estradiol, *P* progesterone, *AMH* anti-Müllerian hormone, *IUI* intrauterine insemination

Data are presented as mean ± SD or number of cases (n) with rate (%)

^a^Value in group C was significantly higher than values in groups A and B. No significant differences between groups A and B

^b^Value in group A was significantly lower than values in group B

^c^Value in group B was significantly higher than values in groups A and C. No significant differences between groups A and C

Table 2 describes the clinical and cycle characteristics of the three groups during IUI treatment. The GnRHa trigger group showed the longest length of LE treatment, and shortest cycle duration among the three groups (*P* < 0.001). Compared with the other two groups, the GnRHa trigger group also presented the most amount of lead follicles, the lowest LH level, and the highest E2 level, but the highest average E2 and the lowest P level (the ratio of E2 to the dominant follicle count) were found in the dual trigger group(*P* < 0.001).

**Table 2:** The cycle characteristics and hormone profiles of patients undergoing IUI

	Group A: 5000 IU hCG (*n* = 1568)	Group B: 0.1 mg GnRHa (*n* = 4034)	Group C: 5000 IU hCG + 0.1 mg GnRHa (*n* = 473)	*P* value
Total LE dose per cycle (mg)	15.43 ± 6.16	15.52 ± 5.76	15.29 ± 7.05	0.730
Length of LE treatment (days)				< 0.001
3	282 (18.0)	747 (18.5)	96 (20.3)	
4	326 (20.8)	590 (14.6)	240 (50.7)	
5	960 (61.2)	2697 (66.9)	137 (29.0)	
Cycle duration (days)	11.26 ± 2.62	10.89 ± 2.65	11.10 ± 2.75	< 0.001^a^
Endometrial thickness at triggering (mm)	9.84 ± 2.26	9.96 ± 2.24	9.81 ± 2.25	0.125
Hormone level on trigger day				
E_2_(pg/ml)	237.77 ± 141.81	281.46 ± 197.58	244.92 ± 121.47	< 0.001^b^
Average E_2_(pg/ml)	183.07 ± 97.65	190.43 ± 137.37	207.20 ± 104.35	< 0.001^c^
LH (mIU/mL)	16.25 ± 14.45	10.76 ± 10.44	13.09 ± 12.18	< 0.001^d^
P (ng/mL)	0.50 ± 0.38	0.42 ± 0.34	0.40 ± 0.31	< 0.001^e^
No. of lead follicles				< 0.001
1	1050 (67.0)	2112 (52.4)	357 (75.5)	
2	409 (26.1)	1360 (33.7)	99 (20.9)	
3	109 (7.0)	562 (13.9)	17 (3.6)	
Lead follicle size, n (%)				0.892
14.1–16.0 mm	27 (1.7)	67 (1.7)	6 (1.3)	
16.1–18.0 mm	131 (8.4)	337 (8.4)	43 (9.1)	
18.1–20.0 mm	606 (38.6)	1630 (40.4)	188 (39.7)	
20.1–22.0 mm	804 (51.3)	2000 (49.6)	236 (49.9)	

*Abbreviations*: *LE* letrozole, *E*_*2*_ estradiol, *Average E*_*2*_ the ratio of E_2_ to dominant follicles count, *LH* luteinizing hormone, *P* progesterone

Data are presented as mean ± SD or number of cases (n) with rate (%)

^a^Value in group B was significantly lower than value in groups A

^b^Value in group B was significantly higher than values in groups A and C. No significant differences between groups A and C

^c^Value in group C was significantly higher than values in groups A and B. No significant differences between groups A and B

^d^Value in group A was significantly higher than values in groups B and C. Value in group C was significantly higher than value in group B

^e^Value in group A was significantly higher than values in groups B and C. No significant differences between groups B and C

The pregnancy outcomes are shown in Table 3. Patients had similar rates of clinical pregnancy, biochemical pregnancy, ongoing pregnancy, and live birth among the three groups. Additionally, no difference in the rates of ectopic pregnancy, multiple pregnancy, and triplet was observed. However, notably, the miscarriage rate was significantly lower in the GnRHa trigger group, and higher in the dual trigger group compared with the hCG group(*P* = 0.045).

**Table 3:** The pregnancy outcomes of patients undergoing IUI

	Group A: 5000 IU hCG (*n* = 1568)	Group B: 0.1 mg GnRHa (*n* = 4034)	Group C: 5000 IU hCG + 0.1 mg GnRHa (*n* = 473)	*P* value
Clinical pregnancy	247 (15.8)	647 (16.0)	75 (15.9)	0.964
Biochemical pregnancy	274 (17.5)	704 (17.5)	83 (17.5)	0.999
Ongoing pregnancy	209 (13.3)	586 (14.5)	64 (13.5)	0.475
Live birth	202 (12.9)	567 (14.1)	61 (12.9)	0.456
Ectopic pregnancy	7/247 (2.8)	9/647 (1.4)	0	0.179
Multiple pregnancy	20/247 (8.1)	56/647 (8.7)	4/75 (5.3)	0.609
Triplet	0	1/647 (0.2)	0	1.000
Miscarriage	45/247 (18.2)	80/647 (12.4)	14/75 (18.7)	0.045^a^

Data are presented as cases (n) with rate (%)

^a^*P* < 0.05

Table 4 demonstrates the impact of the factors that may affect clinical pregnancy outcomes in LE-HMG IUI cycles according to multiple binary logistic analysis. Duration of infertility was negatively correlated with clinical pregnancy rate, while cycle duration and endometrial thickness at triggering significantly increased the chance of clinical pregnancy. Regarding the other factors affecting CPR, infertility cause and the number of lead follicles were found to be significant (*P* < 0.05). Although the length of LE treatment, number of antral follicles, and rank of IUI attempts were different among the groups, they had no effect on pregnancy rates. Notably, different triggering strategies also did not affect the clinical pregnancy rate.

**Table 4:** Binary logistic regression to account for variables of interest associated with clinical pregnancies and miscarriage after undergoing letrozoleovulation induction with intrauterine insemination (*n* = 6,075)

	Adjusted OR (95% CI)	*P* value
Clinical Pregnancy		
Cycle duration (days)	1.044 (1.006–1.083)	0.021
Endometrial thickness at triggering (mm)	1.064 (1.024–1.106)	0.002
No. of lead follicles (1 vs. 2)	1.964(1.493–2.584)	< 0.001
No. of lead follicles (1 vs. 3)	2.051 (1.309–3.215)	0.002
Infertility cause (ovulatory dysfunction vs sexual dysfunction)	2.107 (1.463–3.035)	< 0.001
Rank of IUI attempts (1^st^ vs. 2^nd^)	0.848 (0.705–1.018)	0.077
Duration of infertility (years)	0.934 (0.893–0.976)	0.002
5000 IU hCG vs. 0.1 mg GnRHa	1.035 (0.852–1.257)	0.728
5000 IU hCG vs. 5000 IU hCG + 0.1 mg GnRHa	1.147 (0.835–1.576)	0.397
Miscarriage		
Cycle duration (days)	1.130 (1.022–1.249)	0.017
Trigger day LH level (mIU/mL)	1.020 (1.002–1.038)	0.029
Infertility cause (ovulatory dysfunction vs sexual dysfunction)	2.953 (1.059–8.239)	0.039
Rank of IUI attempts (1^st^ vs. 3^rd^)	2.512 (1.093–5.775)	0.030
5000 IU hCG vs. 5000 IU hCG + 0.1 mg GnRHa	0.427 (0.183–0.996)	0.049
0.1 mg GnRHa vs. 5000 IU hCG + 0.1 mg GnRHa	0.298 (0.128–0.693)	0.005

*CI* confidence interval, *OR* odds ratio

Other factors included (not significant): Total LE dose per cycle, Length of LE treatment, Trigger day E2 level, Trigger day average E2 level, Trigger day LH level, Trigger day P level, Female age, Male age, Female BMI, Male BMI, Infertility type, Infertility cause, Antral follicles, Follicle size

To further assess the factors that may affect miscarriage in LE-HMG IUI cycles, another multiple binary logistic analysis was performed (Table 4). Miscarriage was more likely to happen with prolonged cycle duration and higher LH level on the trigger day. Patients with sexual dysfunction were 2.953 times more likely to have miscarriages than those with ovulatory dysfunction. Also, patients going through their third IUI attempt had an increased chance of miscarriage compared with those undergoing their first attempt. It is worth mentioning that the association between triggering strategy and miscarriage found in Table 3 was confirmed by the binary logistic analysis. According to the result, patients using dual trigger might be more likely to experience miscarriage than the ones using hCG or GnRHa.

A total of 830 patients gave live birth in this study. Table 5 presents the maternal characteristics and neonatal outcomes of these patients. No significant differences were observed regarding maternal age, delivery mode, gestational age, birthweight, birth length, and birth defect. The lowest body mass index was found in the GnRHa trigger group (*P* = 0.030). The dual trigger group presented the longest duration of infertility (*P* = 0.036).

**Table 5:** maternal characteristics and neonatal outcomes of patients who gave live birth

	Group A: 5000 IU hCG (*n* = 202)	Group B: 0.1 mg GnRHa (*n* = 567)	Group C: 5000 IU hCG + 0.1 mg GnRHa (*n* = 61)	*P* value
Maternal age (years)	30.31 ± 3.49	30.64 ± 3.53	31.44 ± 3.81	0.090
Body mass index (kg/m^2^)	22.84 ± 3.33	22.27 ± 3.39	23.18 ± 3.59	0.030^a^
Duration of infertility (years)	2.76 ± 1.85	3.00 ± 1.91	3.66 ± 2.63	0.036^b^
Delivery mode, n (%)				0.437
Vaginal delivery	74 (36.6)	237 (41.8)	25 (41.0)	
C-section delivery	128 (63.4)	330 (58.2)	36 (59.0)	
Gestational age, n (%)				0.662
< 28 weeks	0	2 (0.4)	0	
28–37 weeks	22 (10.9)	44 (7.8)	3 (4.9)	
37–42 weeks	179 (88.6)	518 (91.4)	58 (95.1)	
≥ 42 weeks	1 (0.5)	3 (0.5)	0	
Birthweight, n (%)				0.379
< 2500 g	20 (9.9)	39 (6.9)	2 (3.3)	
2500–4000 g	171 (84.7)	485 (85.5)	55 (90.2)	
≥ 4000 g	11 (5.4)	43 (7.6)	4 (6.6)	
Birth length (cm)	49.48 ± 3.05	49.79 ± 2.17	49.90 ± 1.63	0.343
Birth defect, n (%)				
Nervous system	1 (0.5)	1 (0.2)	0	0.537
Circulatory system	1 (0.5)	1 (0.2)	1 (1.6)	0.112
Endocrine system	0	3 (0.5)	0	0.661
Respiratory system	0	1 (0.2)	0	1.000
Genitourinary system	0	1 (0.2)	0	1.000
Congenital malformation	0	2 (0.4)	0	1.000

Data are presented as mean ± SD or number of cases (n) with rate (%)

^a^Value in group B was significantly lower than values in groups A and C. No significant differences between groups A and C

^b^Value in group C was significantly higher than values in groups A and B. No significant differences between groups A and B

## Discussion

In this study, clinical pregnancy rate was generally similar among hCG, GnRHa and dual trigger LE-HMG IUI cycles. However, miscarriage was found to be associated with triggering strategy. Specifically, the miscarriage rate was significantly lower in the GnRHa triggered group, while it was higher in the dual trigger group.

A study by Le et al. found that CPR was lower in IUI cycles that received GnRHa compared to hCG (13.3% vs. 23.2%), but the difference was not significant [17]. Another similar study by Soliman et al. also found no significant difference in CPR between these two groups, but higher pregnancy rate was seen in the GnRHa-trigger group rather than the hCG group (17.61% vs. 13.06%) [16]. Both studies used HMG for ovarian stimulation and gave luteal phase support. Nonetheless, Romeu et al. reported that pregnancy rates were significantly higher in FSH/GnRHa group than in FSH/hCG group (27.25% vs 17.31%, *P* = 0.0007). In our study, there was no significant difference in CPR between hCG, GnRHa, and dual trigger cycles, which supported the findings of Le et al. and Soliman et al. [16, 17]. However, these two studies both use luteal phase support to compensate for the luteal phase deficiency resulting from GnRHa, which is known to cause luteal phase deficiency due to its short half-life and subsequent short duration of LH surge [10, 11]. In our study, we didn’t provide luteal phase support in any of the three groups. Instead, the luteal function was rescued by the usage of LE. LE has been found to improve the luteal function by better stimulation of the corpus luteum and progesterone secretion [21]. In a prospective randomized controlled study, mid-luteal progesterone was significantly higher in the LE group than the CC group [25]. This result confirmed that patients given LE for ovulation stimulation could form adequate corpus luteum function. Thus, although the findings of Le et al. and Soliman et al. look similar to our study, the experimental design and underlying mechanism of the result are much different.

Apart from improving luteal function, LE also has another advantage. LE inhibits the aromatization of androgens to estrogens, which releases the hypothalamic-pituitary axis from estrogenic negative feedback and increases FSH secretion [19]. Studies showed that increased serum androgen level amplifies FSH receptor gene expression, making follicles more sensitive to FSH [26]. In addition, LE avoids cervical mucus and endometrial morphology interaction due to its lack of antiestrogenic effect [19].

Despite the trigger strategy, which had been proven not associated with clinical pregnancy, there were some other factors that affect this indicator in our study. First, the patients diagnosed with sexual dysfunction were more likely to get pregnant than those diagnosed with ovulatory dysfunction, which was in sync with common belief [27]. Secondly, it was observed that the duration of infertility was negatively correlated with CPR. Similarly, Mahnaz et al. reported that the duration of infertility ≤ 5 years was a favorable factor for IUI treatment [28]. Moreover, compared with patients with only one lead follicle at triggering, those with two or three lead follicles were both more likely to succeed after IUI. Merviel et al. reached a similar conclusion to ours [1]. Finally, endometrial thickness on trigger day was found to be positively correlated with CPR. Marhar et al. [29] found a link between endometrial thickness and the pregnancy rate in IUI and IVF cycles. However, Kolibianas et al. [30] demonstrated that endometrial thickness was not a predictive factor during IUI with clomiphene citrate.

It was suggested that miscarriage was associated with the triggering strategy. Specifically, the miscarriage rate was significantly lower in the GnRHa triggered group, while it was higher in the dual trigger group. This topic remains controversial. Humaidan et al. reported higher miscarriage rate in patients used GnRHa compared with hCG [31], which was explained by luteolysis of corpus luteum after GnRHa. Others had opposite results [6, 18]. The flare-up effect of GnRHa can cause a physiological increase of both FSH and LH, which had a positive effect on growing and distribution of the cumulus cell masses [18]. Moreover, the luteal phase steroid levels over the physiological range after continuous stimulation of hCG may suppress the release of endogenous gonadotropins required for corpus luteum support and exhibit a negative impact on endometrial receptivity [6]. Regarding the high miscarriage rate in the dual trigger group, the sample size was much smaller in this group, which might lead to bias in some statistical results. Also, the portion of patients undergoing 2^nd^ and 3^rd^ IUI cycles is significantly higher in this group. Since the choice of triggering strategy was based on physician preference, patients who fail the first cycle were prone to be treated with both GnRHa and hCG. The interaction between cycle rank and trigger strategy might explain the higher miscarriage rate in the dual trigger group, since the 3^rd^ cycles had also been indicated to be correlated with miscarriage in IUI. Other factors affecting the miscarriage rate included cycle duration and infertility cause. Limited studies had been focused on these topics. Also, we found that higher LH level on the trigger day was associated with miscarriage, but Benmachiche et al. reported that low LH level on the day of GnRHa trigger increased early miscarriage rate [32]. Further research is needed in this field.

Regarding the neonatal outcomes, the live-born infants in the three groups presented comparable birthweights, birth lengths, gestational age, delivery mode, and birth defects, suggesting no association between triggering strategies and adverse neonatal outcomes. The results of our study are similar with those of Budinetz et al. [33]. They found no significant differences in congenital anomalies or neonatal complications between the hCG trigger and GnRHa trigger groups. Bonduelle et al. also drew a similar conclusion with ours [34]. These findings provide reassurance for patients regarding the safety of different triggering strategies.

The current study had several limitations and advantages. A major weakness of it is its retrospective and non-randomized design. It would be better if it was a multicenter study. Although logistic regressions were used for correction of some bias, large differences in sample size between groups might still lead to some statistic problems. Another aspect worth improving is that only patients who completed their follow-up were enrolled, which means patients who were lost to follow-up or with cycle cancellations were not included in the scope of this study. Despite these limitations, our study is among the first to investigate the effect of triggering strategy in LE-HMG IUI cycles with large sample size, which is a huge advantage as a retrospective study. Moreover, IUI cycles using a dual trigger in addition to GnRHa and hCG only, which is seldom mentioned in previous studies, was preliminarily evaluated in the present research. Additionally, we observed maternal characteristics and neonatal outcomes of live newborns, the results of which strongly validated the safety of different trigger strategies with a large cohort. It is also important the study was strictly executed according to good clinical practice guidelines. Further large-sample and multi-center RCTs are needed to confirm this conclusion.

## Conclusions

In conclusion, the results of the present study suggest that both GnRHa and dual trigger can be used to trigger ovulation in LE-HMG IUI cycles, but dual trigger must be used with caution. It is necessary to consider whether the advantages outweigh the risk, inconvenience and cost of using both drugs.

## Data Availability

The data underlying this article will be shared on reasonable request to the corresponding author.

## Abbreviations

LE: Letrozole
HMG: Human menopausal gonadotropin
IUI: Intrauterine insemination
Hcg: Human chorionic gonadotrophin
GnRHa: Gonadotrophin-releasing hormone agonist
CC: Clomiphene citrate
FSH: Follicle-stimulating hormone
LH: Luteinizing hormone
E2: Estradiol
P: Progesterone
AMH: Anti-Müllerian hormone
TVS: Transvaginal ultrasound
MC: Menstrual cycle
CPR: Clinical pregnancy rate
AFC: Antral follicle count
BMI: Body mass index
CI: Confidential interval
